# The establishment of an expected concentration reference range for eltrombopag in the individualized treatment of pediatric immune thrombocytopenia

**DOI:** 10.3389/fphar.2025.1597641

**Published:** 2025-08-21

**Authors:** Nan Wang, Shuyue Dong, Yixin Sun, Jingjing Liu, Zhifa Wang, Jingyao Ma, Xiaoling Wang, Runhui Wu, Xiaoling Cheng

**Affiliations:** ^1^ Department of Pharmacy, Beijing Children’s Hospital, Capital Medical University, Beijing, China; ^2^ School of Basic Medicine and Clinical Pharmacy, China Pharmaceutical University, Nanjing, Jiangsu, China; ^3^ Department of Hematology, Beijing Children’s Hospital, Hemophilia Comprehensive Care Center, National Center for Children’s Health, Capital Medical University, Beijing, China; ^4^ Beijing Children’s Hospital, Clinical Research Center, National Center for Children’s Health, Capital Medical University, Beijing, China; ^5^ Department of Pediatric, Beijing Shijitan Hospital of Capital Medical University, Beijing, China

**Keywords:** eltrombopag, immune thrombocytopenia, children, individualized treatment, therapeutic drug monitoring

## Abstract

**Background:**

Differences in the clinical efficacy and adverse drug reactions (ADRs) of eltrombopag (ELT) in children with immune thrombocytopenia (ITP) may be positively correlated with the serum trough concentration of ELT. Individual pharmacokinetic variations primarily contribute to differences in ELT concentration among individuals. This study is the first to establish an expected concentration reference range for ELT in treating pediatric persistent/chronic ITP (P/CITP) across different age-groups.

**Methods:**

A total of 94 patients with 111 serum trough concentrations were analyzed to validate this range. The median age of patients was 7.68 (5.35, 10.21) years, and 44.7% (42/49) of them were male.

**Results:**

Subgroup analyses revealed significant differences in ELT concentration related to the age, efficacy, and ADR occurrence. The expected concentration reference range was determined using the dose-related concentration (DRC) factor combined with the ELT dosage. The DRC factor ranges were as follows: 0.083–0.216 (mg/L)/mg in children aged 1–6 years, 0.058–0.125 (mg/L)/mg in children aged 7–12 years, and 0.043–0.097 (mg/L)/mg in children aged 13–18 years. Among patients with measured trough concentrations within the expected reference range, 84.3% (59/70) achieved response/complete response (R/CR) and 88.6% (62/70) did not experience ADR.

**Conclusion:**

Serum trough concentration monitoring based on the established reference ranges could enhance the precision of individualized ELT therapy in pediatric ITP patients.

## Introduction

Immune thrombocytopenia (ITP) is an acquired autoimmune disease manifesting as decreased platelet counts and hemorrhagic manifestations (Kohli and Chaturvedi, 2019). The pathogenesis of ITP was primarily attributed to both accelerated platelet destruction and compromised production mechanisms (Neunert et al., 2019; Provan et al., 2019). Eltrombopag (ELT), a thrombopoietin receptor agonist (TPO-RA), is the first guideline-recommended second-line treatment for pediatric ITP (Neunert et al., 2019; Provan et al., 2019). ELT acts on the transmembrane structural domain of the thrombopoietin (TPO) receptor and enhances megakaryocyte proliferation and differentiation by enhancing TPO signaling to the downstream JAK/STAT, AKT, and MAPK pathways, thus increasing platelet production (Kim et al., 2018; Gilreath et al., 2021). The efficacy range of ELT in pediatric ITP is 62% ∼ 76.7% (Bussel et al., 2015; Grainger et al., 2015; Chen M. et al., 2021; Cheng et al., 2021; Wang et al., 2023; Yang et al., 2024), whereas the incidence of adverse drug reactions (ADRs) is approximately 20% (Kim et al., 2018; Cheng et al., 2021; Yang et al., 2024; Burness et al., 2016).

Previous studies have shown that these therapeutic differences are positively correlated with ELT blood concentrations (Marano et al., 2018; Zuo et al., 2020; Zuo et al., 2022; Dong et al., 2024a; Dong et al., 2024b). However, individual pharmacokinetic (PK) variations significantly contribute to inter-individual differences in ELT concentration, particularly in pediatric patients (Gibiansky et al., 2011; Hayes et al., 2011; Wire et al., 2018; Dionisi et al., 2021). Factors influencing ELT exposure include race (Gibiansky et al., 2011; Hayes et al., 2011; Wire et al., 2018), age (Hayes et al., 2011; Dionisi et al., 2021), gender (Gibiansky et al., 2011; Dionisi et al., 2021; Chen J. et al., 2021), disease status (Gibiansky et al., 2011; Hayes et al., 2011), single-nucleotide polymorphism (SNP) of drug transporter proteins and cytochrome P450 enzymes (Marano et al., 2018; Zuo et al., 2022; Chen J. et al., 2021), and many other factors.

Despite its widespread application in other therapeutic areas, the use of therapeutic drug monitoring (TDM) in TPO-RAs remains underexplored (Fang et al., 2024). Our previous study demonstrated that adjusting the ELT dosage based on serum trough concentrations improved the response rates and reduced ADR incidence in pediatric ITP patients (Dong et al., 2024a). However, no reference concentration range exists for clinical guidance. This study is the first to employ a pharmacokinetic approach to develop individualized concentration reference ranges for pediatric ITP patients across different age-groups and dosages.

## Methods

### Study design and patients

Children with ITP who used eltrombopag from June 2022 to December 2024 were enrolled in this observational study. This study was approved by the Beijing Children’s Hospital’s Ethics Committee and was conducted in accordance with the Declaration of Helsinki.

Inclusion criteria were as follows: 1) patients who were younger than 18 years with a confirmed diagnosis of ITP, 2) use of eltrombopag for at least 2 weeks with a fixed dose and regular intake, 3) patients monitored for serum concentrations of eltrombopag in our center.

Exclusion criteria were as follows: 1) high suspicion/diagnosis of other causes of thrombocytopenia, 2) abnormalities in the liver and kidney function before enrollment (ALT, AST, total bilirubin, blood creatinine, etc., greater than 1.5 times the upper limit of the normal value), and 3) those who had received any TPO-RAs or recombinant human thrombopoietin (rhTPO) treatment within 30 days before the start of the present study, which was done to avoid potential PK/PD confounding due to the residual effects of prior biologic treatments.

### Expected concentration reference range of eltrombopag

The expected concentration (*C*_exp_) reference range can be expressed as *C*_exp_ ± SD. *C*_exp_ was calculated by the eltrombopag daily maintenance dose (*D*), the apparent total clearance ( $\frac{Cl}{F}$ ), the elimination rate constant (*k*_*e*_) that can be calculated from the elimination half-life ( $\left.t_{\frac{1}{2}}\right)$ by $k_{e}=\frac{\ln2}{t_{\frac{1}{2}}}$ , and the time of blood withdrawal (*∆t*) (Hiemke et al., 2018). The pharmacokinetic parameters $\frac{Cl}{F}$ and $t_{\frac{1}{2}}$ are available from the PETIT trials (Bussel et al., 2015; ClinicalTrials.gov, 2009) and are shown in Formula 1.

$$C_{\exp}=\left[\left(\frac{D}{24}\right)\times \left(\frac{F}{\text{Cl}}\right)\right]\times \left[\frac{k_{e}\times 24}{1‐e^{‐k_{e}\times 24}}\right]\times e^{‐k_{e}\times ∆t}.$$ (1)

The dose-related concentration (DRC) factor was calculated by omitting the dose from Equation 1 to calculate the expected concentrations at different doses, as shown in Formula 2 (Rognstad et al., 2021).

$$\text{DRC factor}=\frac{C_{\exp}}{D}=\left[\left(\frac{1}{24}\right)\times \left(\frac{F}{\text{Cl}}\right)\right]\times \left[\frac{k_{e}\times 24}{1‐e^{‐k_{e}\times 24}}\right]\times e^{‐k_{e}\times ∆t}.$$ (2)

In the outpatient setting, the medication–blood collection interval cannot be accurately controlled to 24 h, so the tested trough concentrations (*C*_*test*_) may be influenced due to the actual interval time. Therefore, we used Formula 3 to correct eltrombopag’s trough concentration (*C*_*24*_) for each patient at 24 h after drug administration.

$$C_{24}=C_{\text{test}}\times e^{‐k_{e}\times \left(24‐t_{\text{test}}\right)},$$ (3)

where *C*_*test*_ is the actual drug concentration measured at *t*_*test*_, *C*_*24*_ is the trough concentration calculated at 24 h, and *k*_*e*_ is the elimination rate constant ( $k_{e}=\frac{\ln2}{t_{\frac{1}{2}}}$ ).

### Measurement of eltrombopag serum concentrations

Blood samples were drawn before medication to test the trough concentration. All samples were drawn via the forearm vein into tubes containing K3-EDTA and were processed immediately after collection. The protein precipitation method was used for sample preparation (Dong et al., 2024a; Dong et al., 2024b). Eltrombopag-^13^C4 was used as the internal standard, and a standard curve was established using the eltrombopag reference standard. The concentration was assayed by Japer high-performance liquid chromatography-tandem mass spectrometry (AB Sciex Triple Quad 4500 MD). The mobile phases were sodium hexanesulfonate (Sinopharm) and acetonitrile (Tedia), with gradient elution. The stock solution was configured by accurately weighing 10.204 mg of the eltrombopag control, adding it in a 10-mL volumetric flask, adding DMSO to dissolve and make up 1,000 μg/mL of the eltrombopag storage solution, and storing it at −80 °C in a refrigerator. The concentration of eltrombopag showed good linearity within the range of 0.1–25 μg/mL. The minimum quantification limit was 0.1 μg/mL. Both intra-day and inter-day precision were less than ±10%. The method recovery rates of eltrombopag at low, medium, and high concentrations ranged from 90% to 100%, and the matrix effect was between 85% and 110%. Analyst^®^ 1.6.3 software was used for method establishment and result analysis. For detailed information, refer to Supplementary Material 1.

### Data collection

Demographic and clinical characteristics were recorded for the age, sex, weight, disease duration, baseline platelet counts, previous ITP medications, concomitant medications, medication dose, treatment duration, trough serum concentration, platelet counts, bleeding scores, adverse reaction, and laboratory data.

### Treatment and outcome

The initial dosage of eltrombopag was 1.5 mg/kg per day for children aged 5 years or younger, 37.5 mg per day for children older than 5 years and weighing <27 kg, and 50 mg per day for children older than 5 years and weighing ≥27 kg (Cheng et al., 2021). The dose titration was adjusted for platelet response and bleeding events. The maximum dose should not exceed 75 mg daily. Eltrombopag was discontinued if the platelet count was ≥400 ×10^9^/L. Each dose adjustment should be observed for at least 2 weeks to evaluate the platelet count fully (Dong et al., 2024b).

The treatment outcomes were assessed based on platelet counts, bleeding events, and adverse drug reactions (ADRs). Platelet counts were measured and recorded for the following responses (Rodeghiero et al., 2009): 1) complete response (CR): complete platelet response was defined as a platelet count ≥100 × 10^9^/L for at least one measurement (at least 7 days after the initiation of eltrombopag and no more than 4 weeks after its initiation) without the requirement for rescue therapy; 2) response (R): platelet response was defined as a platelet count of 30–100 × 10^9^/L, with at least a twofold increase in platelet count from baseline for at least one measurement (at least 7 days after initiation of eltrombopag and no more than 4 weeks after its initiation) without the requirement for rescue therapy; 3) overall response (OR): overall platelet response was defined as the total number of patients achieving CR and R; and 4) no response (NR): no platelet response was defined as a platelet count <30 × 10^9^/L, less than a twofold increase in the baseline count, or bleeding after dose titration of eltrombopag. Bleeding scores were graded by an updated bleeding scale for pediatric patients with ITP: grade 1, minor; grade 2, mild; grade 3, moderate; and grade 4, severe (Provan et al., 2019). Adverse reactions were graded according to the WHO-UMC criteria (WHO, 2025).

### Statistical analysis

Descriptive data summaries and the calculation of pharmacokinetic formulas were carried out using Excel (Microsoft) and R 4.4.1. Continuous variables were described as the mean with standard deviations (SD) or the medians with the interquartile range (IQR), and categorical variables were presented as counts (percentages). Correlation tests were conducted using the Pearson coefficient (*r*_*p*_) for normal-distributed variables, and the Spearman rank correlation coefficient (*r*_*s*_) was applied for non-normal-distributed variables. Group differences were analyzed by the independent two-tailed t-test or Mann–Whitney U test for two continuous variables, one-way ANOVA or the Kruskal–Wallis H-test for multiple continuous variables, and the chi-squared test for categorical variables. Statistical significance was defined as the *p*-value <0.05. Data were analyzed using SPSS 24.0. Figures were drawn using GraphPad Prism 9.0.

## Results

### Baseline characteristics

A total of 94 patients contributed 111 trough concentration measurements. The median age was 7.68 (IQR, 5.35, 10.21) years, with 44.7% (42/94) patients being male. All patients were diagnosed with P/CITP, with 34 cases of PITP and 77 cases of CITP. The median duration of ITP was 23 months (IQR, 11.82, 44.84). The median duration of ELT treatment was 4.67 months (IQR, 0.82, 16.52), and 68.1% (64/94) of children underwent ELT monotherapy. The most commonly used combination drugs were immunosuppressive drugs such as corticosteroids and rituximab. A total of 63.8% (60/94) patients used 3∼5 types of previous ITP medication before ELT treatment, including intravenous immunoglobulin (IVIG), corticosteroids, rituximab, recombinant human TPO (rhTPO), other TPO-RAs, or additional ITP medications. The median platelet count (PLT) at baseline and TDM were 19 (IQR, 12, 28) × 10^9^/L and 75 (IQR, 38.5, 137) × 10^9^/L, respectively. The median baseline TPO level was 67.45 (IQR, 47.85, 90.3) pg/mL. The proportion of megakaryocyte classification in <100/slide, 100 ∼ 300/slide, and >300/slide at baseline were 12.8% (12/94), 33% (31/94), and 54.3% (51/94), respectively. The information about patients’ baseline characteristics is shown in Supplementary Table 2.

### Eltrombopag concentration and subgroup analysis

For all patients, the mean ± SD of the ELT maintenance dose was 30.88 ± 12.75 mg and 1.137 ± 0.447 mg/kg. The mean ± SD of ELT serum trough concentration was 2.729 ± 1.778 mg/L. For patients who achieved R or CR and did not experience any ADRs (N = 70), the mean ELT dose was 29.59 ± 12.43 mg/1.032 ± 0.459 mg/kg, and the mean ELT trough concentration was 2.419 ± 1.120 mg/L. Based on this, a preliminary reference range of serum trough concentrations in children with P/CITP treated with ELT can be obtained as 1.299 ∼ 3.539 mg/L.

**FIGURE 1:**
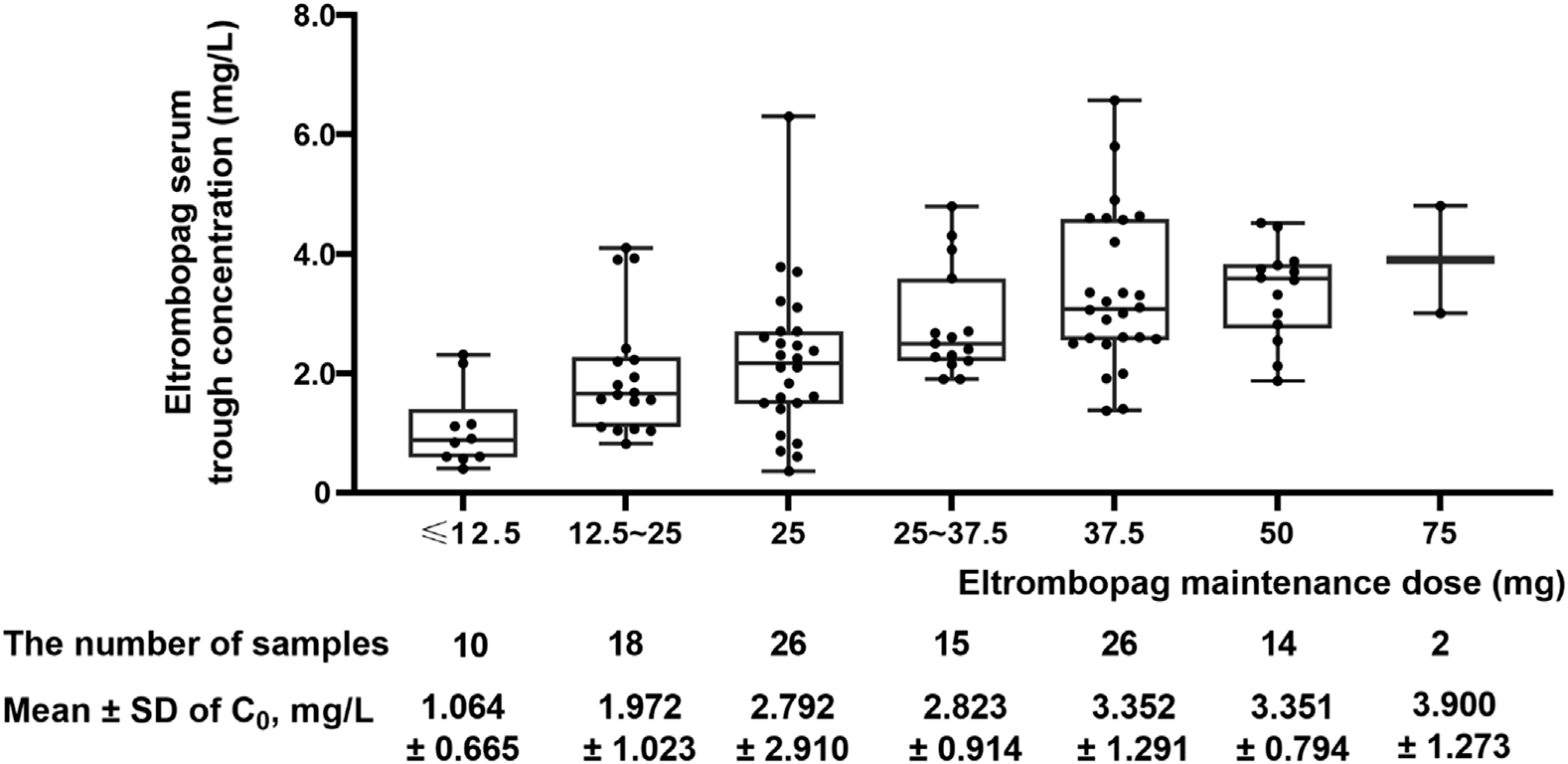
Maintenance dose and serum trough concentration of eltrombopag.

The distributions of ELT maintenance dose and serum trough concentration are shown in Figure 1. To eliminate the effect of the ELT dose on blood concentrations, corrected concentrations were obtained by dividing the concentration of the ELT dose. For pediatric patients, Pearson’s correlation analysis was performed between the daily dose and body weight dose with serum trough concentration: r = 0.585, *p* < 0.001 and r = 0.496, *p* < 0.001. Table 1 presents the results of the maintenance dose, serum trough concentration, and corrected concentrations in subgroup analyses. These subgroup analyses found significant differences in the corrected concentrations related to the age, efficacy, and ADRs.

**TABLE 1:** Eltrombopag maintenance dose and concentrations in different subgroups.

Sample (N)	ELT concentration (mg/L)	*p*_*1*_ value	ELT concentration/daily dose (mg/L)/mg	*p*_*2*_ value	ELT concentration/weight dose (mg/L)/(mg/kg)	*p*_*3*_ value
All (111)	2.729 ± 1.778	—	0.093 ± 0.066	—	2.521 ± 1.283	—
Aged 1–6 years (41)	2.776 ± 2.393	0.564	0.115 ± 0.098	0.029	1.898 ± 1.041	<0.001
Aged 7–12 years (57)	2.761 ± 1.341		0.086 ± 0.032		2.709 ± 1.141	
Aged > 12 years (13)	2.440 ± 1.274		0.062 ± 0.027		3.614 ± 1.607	
Boys (51)	2.567 ± 2.198	0.378	0.095 ± 0.088	0.726	2.457 ± 1.448	0.758
Girls (60)	2.868 ± 1.317		0.091 ± 0.041		2.577 ± 1.130	
PITP (34)	3.028 ± 2.493	0.249	0.105 ± 0.102	0.192	2.300 ± 1.281	0.153
CITP (77)	2.600 ± 1.365		0.087 ± 0.041		2.616 ± 1.280	
Monotherapy (79)	2.613 ± 1.341	0.277	0.088 ± 0.043	0.276	2.482 ± 1.225	0.839
Combination therapy (32)	3.024 ± 2.586		0.104 ± 0.104		2.620 ± 1.436	
ELT treatment time < 3 months (52)	3.055 ± 2.088	0.100	0.102 ± 0.087	0.383	2.450 ± 1.191	0.086
ELT treatment time 3∼12 months (27)	2.745 ± 1.362		0.083 ± 0.043		2.200 ± 1.056	
ELT treatment time > 12 months (32)	2.195 ± 1.436		0.086 ± 0.035		2.907 ± 1.520	
NR (29)	2.421 ± 1.100	0.426	0.085 ± 0.048	0.575	1.845 ± 0.868	0.001
R (47)	2.712 ± 1.418		0.091 ± 0.046		2.556 ± 1.118	
CR (35)	3.006 ± 2.514		0.102 ± 0.096		3.036 ± 1.527	
ADR: yes (14)	4.873 ± 3.403	<0.001	0.158 ± 0.144	<0.001	3.594 ± 1.494	0.001
ADR: no (97)	2.416 ± 1.125		0.083 ± 0.039		2.365 ± 1.178	

Notes: concomitant therapy included eight samples with corticosteroids, 11 with rituximab, seven with mineral supplements, and six with traditional Chinese medicine. ADRs were observed in six cases with thrombocytosis, four cases with elevated transaminase, and four cases with elevated alkaline phosphatase (ALP).

Abbreviations: ELT, eltrombopag; PITP, persistent immune thrombocytopenia; CITP, chronic immune thrombocytopenia; NR, no response; R, response; CR, complete response; ADRs, adverse drug reactions. The *p1* value is the group differences in eltrombopag concentrations. The *p2* value is the group difference in ELT correction concentration, which was calculated by concentration/daily dose. The *p3* value is the group difference in ELT correction concentration, which was calculated by concentration/weight dose.

### Expected concentration reference range of eltrombopag

Unlike adults, the pharmacokinetic characteristics of children in different age stages are very different. Therefore, we grouped children according to their age and the dose of ELT used. Pharmacokinetic parameters were obtained from the PETIT trial (Bussel et al., 2015; ClinicalTrials.gov, 2009). Table 2 demonstrates the pharmacokinetic parameters for the different age-groups, the upper and lower limits of the DRC factor, and the expected concentrations at the different doses of ELT. The results show that the ranges of the DRC factor were 0.083 ∼ 0.216 (mg/L)/mg in children aged 1–6 years old, 0.058 ∼ 0.125 (mg/L)/mg in children aged 7–12 years old, and 0.043 ∼ 0.097 (mg/L)/mg in children aged 13–18 years old.

**TABLE 2:** Dose-related concentration factor and expected serum concentration ranges.

Age-group (years)	$\frac{Cl}{F}$ ± SD^6^ (mL/h)	$t_{\frac{1}{2}}$ (h)	*∆t* (h)	$k_{e}$	Dose (mg)	Low DRC factor (mg/L)/mg	High DRC factor (mg/L)/mg	Low C_exp_ limit (mg/L)	High C_exp_ limit (mg/L)
1–6	0.25 ± 0.111	26.5	24	0.026	12.5	0.083	0.216	1.036	2.697
					25.0			2.073	5.394
					37.5			3.109	8.091
					50.0			4.146	10.788
					75.0			6.218	16.182
7–11	0.38 ± 0.140	26.5	24	0.026	12.5	0.058	0.125	0.720	1.558
					25.0			1.441	3.116
					37.5			2.161	4.674
					50.0			2.881	6.231
					75.0			4.322	9.347
13–18	0.5 ± 0.192	26.5	24	0.026	12.5	0.043	0.097	0.541	1.215
					25.0			1.082	2.431
					37.5			1.623	3.646
					50.0			2.164	4.862
					75.0			3.246	7.292

Abbreviations: $\frac{Cl}{F}$ , apparent total clearance; data taken from the PETIT trials (Bussel et al., 2015; ClinicalTrials.gov, 2009).

SD, standard deviation for $\frac{Cl}{F}$ , reported in the PETIT trials (Bussel et al., 2015; ClinicalTrials.gov, 2009).

$t_{\frac{1}{2}}$ , drug half-life; *∆t*, medication–blood collection intervals; $k_{e}$ , the elimination rate constant; DRC factor, the dose-related concentration factor; *C*_exp_, expected serum trough concentration, which was calculated by pharmacokinetic Formula 1.

Combining the DRC factor with the ELT dose that each patient received, we calculated all patients’ expected ELT concentration ranges and compared them with their tested serum trough concentration. The results showed that 63.1% (70/111) were in their ranges, 27% (30/111) were below the lower limit, and 9.9% (11/111) were above the higher limit.

We further analyzed and compared this distribution result with ELT efficacy and ADR, and the results were statistically significant (*p* < 0.05), as shown in Table 3. Among patients whose trough concentrations fell within the expected reference range, 84.3% (59/70) achieved R/CR, whereas 88.6% (62/70) remained free of ADRs. For patients whose trough concentrations were below the reference range, only 43.3% (13/30) achieved R/CR, yet 96.7% (29/30) did not experience ADRs. For patients with measured results above the expected reference range, 90.9% (10/11) achieved R/CR and 54.5% (6/11) did not experience ADRs. This result suggests that ELT serum trough concentrations within the expected concentration reference range can achieve efficacy while reducing the incidence of ADRs.

**TABLE 3:** Comparison of *C*_*test*_ and *C*_exp_ ranges with efficacy and ADR.

Comparison of *C*_*test*_ and *C*_exp_ ranges	Efficacy			*p-value*
	NR (29)	R (47)	CR (35)	
Within expected range (70)	11	33	26	0.001*
Below the lower limit (30)	17	7	6	
Above the upper limit (11)	1	7	3	

Comparison of *C*_*test*_ and *C*_exp_ ranges	ADR		*p-value*
	No (97)	Yes (14)	
Within expected range (70)	62	8	0.003*
Below the lower limit (30)	29	1	
Above the upper limit (11)	6	5	

Notes: In *within expected range* group, including 41.5% (17/41) of patients aged 1–6 years, 75.4% (43/57) of patients aged 7–12 years, and 76.9% (10/13) of patients aged >12 years. *Below the lower limit* group included 48.8% (20/41) of patients aged 1–6 years, 14.0% (8/57) of patients aged 7–12 years, and 15.4% (2/13) of patients aged >12 years. *Above the upper limit* group included 9.8% (4/41) of patients aged 1–6 years, 10.5% (6/57) of patients aged 7–12 years, and 7.7% (1/13) of patients aged >12 years.

Abbreviations: *C*_*test*_, serum trough concentrations test in our study; *C*_exp_, expected serum trough concentration, which was calculated by pharmacokinetic formula; NR, no response; R, response; CR, complete response; ADRs, adverse drug reactions.

## Discussion

The effectiveness and safety of ELT in the treatment of ITP in children have been demonstrated in many studies (Neunert et al., 2019; Provan et al., 2019; Bussel et al., 2015; Grainger et al., 2015). In our previous studies, the efficacy of ELT in treating P/CITP in children was approximately 70%, and the incidence of ADRs was approximately 20% (Cheng et al., 2021; Wang et al., 2023). TDM could serve as a valuable tool for optimizing individualized ELT therapy in pediatric P/CITP, balancing efficacy and safety (Dong et al., 2024a; Lo, 2024). However, in our previous study, ELT concentrations higher than 4.33 mg/L were susceptible to ADRs, but the relationship between efficacy and concentration was inconclusive (Dong et al., 2024b). More detailed influencing factors and therapeutic concentration ranges need to be identified.

To better determine the factors affecting ELT concentrations, we introduced corrected concentrations for further analysis. The results showed that the corrected concentrations were significantly different in three subgroups, namely, age, efficacy, and ADR, whereas there were no significant differences in gender, duration of ITP, duration of ELT treatment, and combination of drugs. In pediatric patients, apparent clearance increases with age and weight, leading to reduced ELT exposure (Wire et al., 2018). Therefore, this difference because of the children’s age should be considered when discussing ELT concentrations.

Although there were no statistically significant differences in the ELT treatment time subgroup, we found that patients who exceeded 12 months of ELT treatment had the lowest therapeutic doses but higher corrected concentrations. Most of these patient populations achieved a therapeutic response (R/CR) and were in the drug reduction phase, resulting in lower doses and trough concentrations of ELT than other groups. As the corrected concentration is independent of the drug dose, we speculate that the level of the corrected concentration may serve as a basis for the development of an individualized ELT reduction protocol for children with ITP. Further research is needed in the future to confirm this speculation.

We further compared the relationship between efficacy and ELT concentration. Both the ELT trough concentration and corrected concentration showed that CR > R > NR. This trend is consistent with previous reports (Zuo et al., 2020; Dionisi et al., 2021). However, only the bodyweight corrected concentration had a statistically significant difference (*p* = 0.001), which means patients who used a 1 mg/kg dose of ELT had a higher concentration, corresponding to an increased ELT response rate. Monitoring the correct bodyweight concentration of ELT may help physicians determine whether a patient’s ELT dose is adequate and can provide a reference for patient dose titration to shorten the time for reaching the first response.

ELT treatment in children with ITP was well tolerated, and no serious ADRs were observed in this study. Most ADRs could be relieved by reducing or discontinuing ELT. The serum trough concentration and corrected concentration of ELT were higher in the group that experienced ADRs, suggesting that the higher the exposure to ELT, the higher the risk of ADRs. This finding is consistent with previous studies, and ADRs may be mitigated through ELT concentration monitoring (Marano et al., 2018; Zuo et al., 2022; Dong et al., 2024a).

The expected serum concentration ranges were first introduced to determine ELT’s therapeutic scope for treating children with ITP. Considering the differences in the pharmacokinetic characteristics of children at different ages, the expected concentration reference range was established based on different age-groups. The relevant parameters in the pharmacokinetic formula were obtained in the PETIT/PETIT-2 study (Bussel et al., 2015; Grainger et al., 2015; Wire et al., 2018). The advantage of the DRC factor is that the expected concentration at any dose can be calculated. Based on this, we calculated the expected concentration ranges for each patient in our study and compared their measured results with their own expected serum concentration ranges. A total of 63.1% (70/111) of samples were in the expected range, 27.0% (30/111) of samples were below the range, and 9.9% (11/111) of samples were above the range. Due to ethnic or inter-population pharmacokinetic differences, the apparent clearance of eltrombopag in East-Asian pediatric patients is lower than that in non-East-Asian children (Kim et al., 2018). Additionally, a small percentage of East-Asian children participated in the PETIT study (Bussel et al., 2015). Therefore, the expected concentration range calculated from the pharmacokinetic parameters of the PETIT study should be higher than the measured results. However, our findings indicate that nearly one-third of the patients fell below the lower limit.

We further analyzed the results of the measured concentration outcomes with the expected range of patients in the three age-groups and found that for patients aged 1–6 years, 41.5% (17/41) were the within the expected range group, 48.8% (20/41) were in the below the lower limit group, and 9.8% (4/41) of patients were in the above the upper limit group. Meanwhile, the percentage of patients aged 7–12 years and >12 years in the within the expected range group was 75.4% and 76.9%, respectively. In the PETIT study, children aged 1–6 years were using ELT powder for oral suspension (PfOS), and the bioavailability of PfOS was 22% higher than that of tablets (Wire et al., 2018), which may be the main reason for the higher range of expected concentrations. Due to the accessibility of medicines in China, our study used ELT tablets (25 mg/tablet) for all patients. Children aged 1–6 years usually had to break the dose, which may have resulted in lower bioavailability of ELT and lower measured concentrations. Unfortunately, there are no large, authoritative population pharmacokinetic studies of ELT for treating Chinese children with ITP. Instead, it is recommended that PK parameters for calculating the expected concentration reference range should be derived from the PK study in the same ethnic population and the same dosage form.

In addition, due to the nature of real-world studies, the interval of medication–blood collection could not be precisely controlled at 24 h, which may affect the results of monitoring trough concentrations. The interval of medication–blood collection was 27.99 ± 9.48 h in patients aged 1–6 years, which was longer than that in children aged 7–12 years (26.41 ± 3.38 h) and >12 years (27.09 ± 1.93 h). Although there was no statistical difference in the time interval among the three age-groups, we tried to correct the interval to 24 h using the pharmacokinetic formula to further analyze the effect of inconsistent time intervals. The results suggested that in the age-group of 1–6 years, the concentration in children in the within the expected range subgroup was elevated to 51.2% (21/41), the concentration in children in below the lower limit subgroup was reduced to 39.0% (16/41), and the concentration in children in the above the upper limit group remained at 9.8% (4/41), as shown in Supplementary Table 3.

Combining the efficacy and ADR assessments, 84.3% (59/70) of samples in the within the expected range group achieved R/CR, and 88.6% (62/70) had no ADRs. Patients whose concentration results are within the expected reference range are more likely to achieve a therapeutic response and have a higher therapeutic safety. In practice, the expected concentration reference ranges may serve as targets during ELT titration to optimize the efficacy and minimize ADRs, enabling individualized treatment for children with ITP.

There are some limitations to our study. (1) Small sample size: although this study is a prospective real-world study conducted in a pediatric patient population, for exploring the therapeutic window, a larger sample size is still needed for its validation. (2) Influential factor analysis included fewer factors for discussion. Factors that may be related to the *in vivo* exposure of the drug and its degree of metabolism were not included due to clinical realities. For example, factors such as platelet antibody type and titer, patients’ liver drug enzymes, and their transporter gene polymorphism results were not included. (3) The pharmacokinetic parameters quoted in this study do not accurately reflect the *in vivo* metabolism of ELT in Chinese pediatric ITP patients. Tablet splitting for children aged 1–6 years in the study was the main reason for its low bioavailability. This should be considered in future studies. Although there is a lack of direct, large-scale pharmacokinetic studies on the use of ELT in East Asian pediatric ITP patients, the expected concentration reference ranges calculated from the PETIT study can still provide a reference for pediatric ITP patients.

In conclusion, monitoring the serum trough concentration with the expected reference ranges of ELT could improve the individualized treatment effect in children with ITP.

Method limitation: a concentration-dependent matrix effect was observed, with the most pronounced ion enhancement observed at the LLOQ (0.1 μg/mL). Despite effective reduction of matrix interference through optimized sample preparation and the use of an isotopically labeled internal standard, this residual effect at low concentration may represent a limitation of the method.

## Data Availability

The data analyzed in this study are subject to the following licenses/restrictions: Due to the nature of this research, the participants did not agree that their data should be shared publicly; therefore, supporting data is unavailable. Further inquiries can be directed to the corresponding authors. Requests to access these datasets should be directed to Xiaoling Cheng, chengxiaoling1224@163.com.
