# Supplementary material for: The establishment of an expected concentration reference range for eltrombopag in the individualized treatment of pediatric immune thrombocytopenia
Source: Front Pharmacol. 2025 Aug 21;16:1597641. doi: 10.3389/fphar.2025.1597641 (PMC12408700; doi:10.3389/fphar.2025.1597641)
Supplement: Supplementary file 1 [file Supplementaryfile1.docx]

**Supplementary Material 1 Measurement of eltrombopag serum concentrations**

The precision, accuracy and matrix effect of eltrombopag (n=6, x±s)

| Samples | Analyte QC level（μg·mL^-1^） | intra-day | | | inter-day | | | Recovery% | Matrix factor |
| --- | --- | --- | --- | --- | --- | --- | --- | --- | --- |
|  |  | Measured value（μg·mL^-1^） | CV% | RE% | Measured value（μg·mL^-1^） | CV% | RE% |  |  |
| LLOQ | 0.1 | 0.10±0.01 | 7.95 | 4.18 | 0.11±0.01 | 9.36 | 6.25 | 93.58 | 2.60 |
| L-QC | 0.25 | 0.22±0.01 | 4.58 | -12.00 | 0.22±0.02 | 8.15 | -12.00 | 91.20 | 0.93 |
| M-QC | 7.5 | 7.93±0.42 | 5.30 | 5.73 | 7.82±0.39 | 4.99 | 4.27 | 105.67 | 1.01 |
| H-QC | 20 | 21.13±0.26 | 1.23 | 5.65 | 20.66±0.58 | 2.81 | 3.30 | 105.67 | 1.00 |

LLOQ: Lower Limit of Quantification；L-QC: Low Quality Control；M-QC: Medium Quality Control；H-QC: High Quality Control；RE%：Relative Error (%)；CV%：Coefficient of Variation (%)


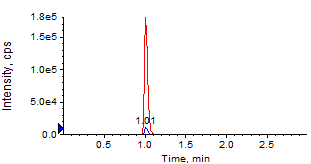


The chromatogram of LLOQ spiked with the internal standard.

（The blue one is the chromatogram of the analyte eltrombopag, and the red one is the chromatogram of the internal standard.）


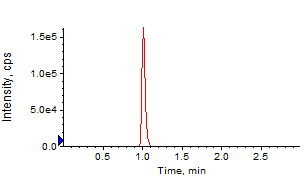


The chromatogram of the blank sample alongside with the internal standard.

(the red one is the chromatogram of the internal standard)

**Supplementary Table 2 Baseline characteristics**

| **Contents** | **Patients (n=94)** |
| --- | --- |
| Age, median (IQR), years | 7.68 (5.35, 10.21) |
| Male, n (%) | 42（44.7%） |
| Weight, median (IQR), kg | 26.6 (19.9, 40.0 ) |
| ITP duration, median (IQR), months | 23 (11.82, 44.84) |
| ELT treatment, median (IQR), months | 4.67 (0.82, 16.52) |
| Baseline platelet count, median (IQR), × 10^9^ /L | 19 (12, 28) |
| Platelet count at TDM time, median (IQR), × 10^9^ /L | 75 (38.5, 137) |
| TPO, median (IQR), pg/mL | 67.45 (47.85, 90.3) |
| Megakaryocytes, n (%)  < 100 /slide  100~300 /slide  > 300 /slide | 12 (12.8%)  31 (33%)  51 (54.3%) |
| ELT monotherapy, n (%) | 64 (68.1%) |
| ITP co-medication, n (%) | 30 (31.9%) |
| Previous ITP treatment, n (%)  < 3 types  3~5 types  > 6 types | 33 (35.1%)  60 (63.8%)  1 (1.1%) |

Abbreviations: ITP, immune thrombocytopenia; ELT, eltrombopag, TDM, therapeutic drug monitoring, TPO, thrombopoietin.

**Supplementary Table 3 Comparison of *C_24_* and *C_exp_* ranges with efficacy and ADR**

|  | | **Efficacy** | | | *p value* |
| --- | --- | --- | --- | --- | --- |
|  |  | NR (29) | R (47) | CR (35) |  |
| Comparison of *C_24_* and *C_exp_* ranges | Within expected range (67) | 13 | 31 | 23 | 0.001* |
|  | Below the lower limit (26) | 15 | 7 | 4 |  |
|  | Above the upper limit (18) | 1 | 9 | 8 |  |

|  | | **ADR** | | *p value* |
| --- | --- | --- | --- | --- |
|  |  | No (97) | Yes (14) |  |
| Comparison of *C_24_* and *C_exp_* ranges | Within expected range (67) | 61 | 6 | 0.002* |
|  | Below the lower limit (26) | 25 | 1 |  |
|  | Above the upper limit (18) | 11 | 7 |  |

Notes: In *Within expected range* group, including 51.2% (21/41) of patients aged 1-6 years, 64.9% (37/57) of patients aged 7-12 years, and 69.2% (9/13) of patients >12 years. In *Below the lower limit* group, including 39.0% (16/41) of patients aged 1-6 years, 14.0% (8/57) of patients aged 7-12 years, and 15.4% (2/13) of patients >12 years. In *Above the upper limit* group, including 9.8% (4/41) of patients aged 1-6 years, 21.1% (12/57) of patients aged 7-12 years, and 15.4% (2/13) of patients >12 years

Abbreviations: *C_24_*, the 24-hour steady-state serum trough concentration, calculated by the pharmacokinetic formula (3). *C_exp_*, expected serum trough concentration, calculated by pharmacokinetic formula. NR, no response. R, response. CR, complete response. ADR, adverse reactions.
